# E3 Ubiquitin Ligases: The Operators of the Ubiquitin Code That Regulates the RLR and cGAS-STING Pathways

**DOI:** 10.3390/ijms232314601

**Published:** 2022-11-23

**Authors:** Likai Ji, Yan Wang, Liying Zhou, Juan Lu, Siwen Bao, Quan Shen, Xiaochun Wang, Yuwei Liu, Wen Zhang

**Affiliations:** School of Medicine, Jiangsu University, Zhenjiang 212013, China

**Keywords:** E3 ubiquitin ligases, RLR, cGAS-STING, virus, autophagy

## Abstract

The outbreaks caused by RNA and DNA viruses, such as SARS-CoV-2 and monkeypox, pose serious threats to human health. The RLR and cGAS-STING pathways contain major cytoplasmic sensors and signaling transduction axes for host innate antiviral immunity. In physiological and virus-induced pathological states, the activation and inactivation of these signal axes are tightly controlled, especially post-translational modifications (PTMs). E3 ubiquitin ligases (E3s) are the direct manipulator of ubiquitin codons and determine the type and modification type of substrate proteins. Therefore, members of the E3s family are involved in balancing the host’s innate antiviral immune responses, and their functions have been extensively studied over recent decades. In this study, we overviewed the mechanisms of different members of three E3s families that mediate the RLR and cGAS-STING axes and analyzed them as potential molecular targets for the prevention and treatment of virus-related diseases.

## 1. Introduction

It is important to protect against emerging and re-emerging viruses by understanding how they successfully evade the immunity systems of humans. Our innate immunity systems are physiologically programmed to be the first line of defense against RNA and DNA virus infections. Several pattern recognition receptors (PRRs) and some nucleic acid sensors have been identified that sense DNA or RNA viruses and initiate innate antiviral immune responses. (i) The retinoic acid-inducible gene-I (RIG-I)-like receptors (RLRs) are the major cytoplasmic PRRs that recognize RNA viruses. RIG-I and melanoma differentiation-associated 5 (MDA5) are the main RLR sensors for recognizing positive and negative ssRNA viruses [1]. The RIG-I/MDA5-MAVS-TBK1-IRF3 axis has been clearly identified as the signal transduction pathway that activates type I IFN production. (ii) The cyclic GMP-AMP synthase (cGAS) has been proven to be a major cytosolic dsDNA sensor [2,3]. STING (the stimulator of the IFN gene, which is also known as MITA, MPYS, TREM173, and ERIS) is the major cGAMP receptor that induces type I IFN production. The cGAS-STING-TBK1-IRF3 axis has been identified as the major mechanism that responds to DNA virus infections. (iii) Toll-like receptors (TLRs) are important membrane-bound PRRs that are distributed in plasma and endosomal membranes to sense pathogen infections [4]. Following PAMPs detection by various PRRs, the complex signal transduction axis is finely regulated to avoid immune dysregulation.

**Figure 1:**
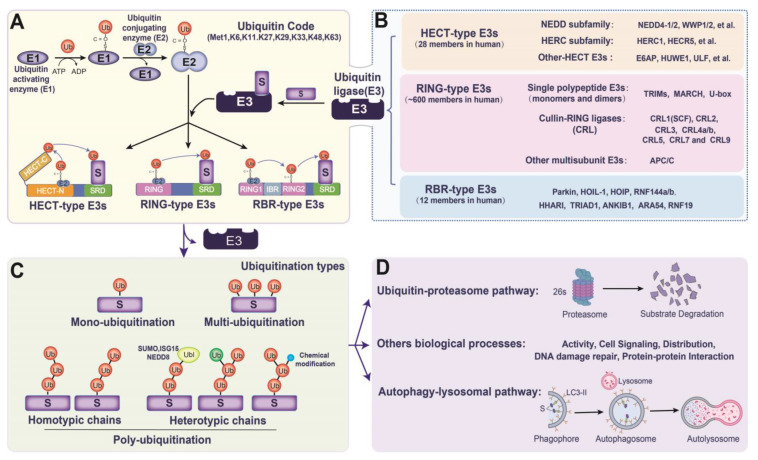
The types and functions of the E3s family: (**A**,**B**) the process of ubiquitin being activated and translocated to a substrate via E1, E2, and E3s (the E3s family is divided into three types (HECT-type, RING-type, and RBR-type E3s), depending on how ubiquitin is transferred from E2 to substrate proteins); (**C**) E3s catalyze substrates to undergo monoubiquitylation, multiubiquitylation or polyubiquitylation; (**D**) ubiquitinated substrates activate proteins, participate in cell signaling, repair DNA damage and distribute and degrade proteins via the ubiquitin-proteasome pathway (UPP) or autophagy-lysosome pathway (ALP). Abbreviations: S, substrate for ubiquitylation; Ub, ubiquitin; HECT, homologous to E6-AP carboxyl terminus; RBR, RING-between-RING; IBR, in-between RING; RING2: RING or Rcat; SRD, substrate-recognizing domain.

Ubiquitylation is one of the most important and pervasive mechanisms and plays an important role in the immune system. Ubiquitin (Ub) is a small protein that is made of 76 amino acids and contains seven lysine sites (K6, K11, K27, K29, K33, K48, and K63), as well as a Met1 site for Ub chain elongation. Ub is activated and transferred to substrate proteins by Ub-activating enzymes (E1s), Ub-conjugating enzymes (E2s), and E3 Ub ligases (E3s) [5]. Ubiquitylated proteins are used to mediate cell signaling, DNA damage repair, and degrade proteins via the ubiquitin-proteasome pathway (UPP) or autophagolysosomal pathway (ALP) (Figure 1). E3s are direct operators that sense and interact with special substrates. In recent decades, extensive research has focused on the mechanisms by which E3s manipulate the Ub-encoded regulation of host innate immunity. Nonetheless, the understanding of the different types of Ub ligation that are mediated by E3s in different substrates is still limited, especially in virus infections. E3 can also be hijacked by viruses to evade host immune surveillance. Therefore, in the present brief review, we focused on the mechanisms of the E3-induced ubiquitylation of substrates.

## 2. Overview of E3s in Ub Transformation

Ubiquitylation is the process through which Ub is activated and transformed into substrates (Figure 1A). Ub monomers are first activated by E1 via ATP to form Ub-adenylate. The activated Ub is then transferred to E2 via thioester bonds. E3s mediate the transfer of Ub from E2 to free amine groups, either at the NH2-terminus of the Ub protein or the internal lysine residue of the substrate protein, resulting in monoubiquitylation, multiubiquitylation, or polyubiquitylation of the substrate protein (Figure 1A,C).

The E3s family in mammals has more than 600 members. According to the ubiquitylation domains and the mechanism of ubiquitin transformation, E3s are roughly divided into three families: RING-type (really interesting new gene), HECT-type (homologous to the E6-AP carboxyl terminus), and RBR-type (RING-between-RING or Rcat) (Figure 1B). RING-type E3s make up the largest E3s subfamily. They are characterized by one or two conserved RING finger motifs and include three subfamilies: single polypeptide E3 (monomer and dimer), Cullin-RING ligases (CRLs), and other multisubunit E3s [5]. RING-type E3s act as a scaffold, orienting the Ub-bearing E2 to the substrates (Figure 1A). Members of the RING finger subfamily contain two-atom zinc cross-support structures, which catalyze the direct transformation of ubiquitin from E2 to the substrates. Unlike RING-type E3s, HECT-type E3s first form an E3-ubiquitin thioester intermediary through their HECT domain, which loads ubiquitin onto itself and then directly catalyzes the ubiquitylation of substrates. The N-terminal region of HECT-type E3s determines their interactions with various substrates. Based on their N-terminal properties, HECT-type E3s can also be divided into three subdivisions: Nedd4-like, HECT and RCC1-like domain (HERC), and “other” HECTs (Figure 1B). RBR-type E3s are the latest subfamily to be isolated from the E3s family. Although they contain a RING domain, they are more similar to HECT-type E3s than RING-type E3s in terms of their mechanism of catalyzing substrate ubiquitylation(Figure 1A). RBR-type E3s maintain an autoinhibited state through intramolecular interactions in the absence of substrate binding. In humans, RBR-type E3s contain conserved RING1, benign-catalytic (BRcat, also named IBR), and Rcat domain. The RING and required-for-catalysis (Rcat) domains of RBR-type E3s are both catalytic domains that are commonly designated RING2 (Figure 1A) [6]. Once autoinhibition is released, RING1 recruits ubiquitin-bound E2, loads ubiquitin into the RING2 domain, and then transfers it directly to the substrate (Figure 1A).

## 3. Manipulation of RING-Type E3s in the RLR and cGAS-STING Pathways

### 3.1. Manipulation of RING-Type E3s That Enhances the RLR and cGAS-STING Pathways

**Figure 2:**
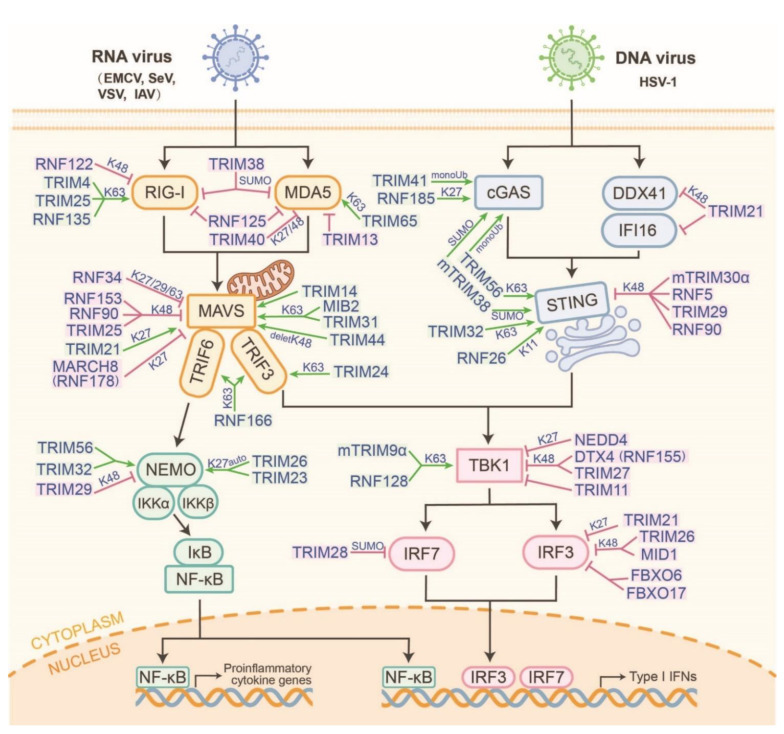
The comprehensive networks of RING-type E3s that regulate the RLR and cGAS-STING pathways. According to the mechanism of RING-type E3s regulatory function, four modes are classified as following: RING-type E3s directly target receptor proteins or act as intermediaries between signal transduction molecules and ubiquitin (e.g., TRIM25, RNF65, RNF185, etc.). RING-type E3s first perform auto-ubiquitylation and then interact with substrates via a signal transduction pathway (e.g., TRIM26). RING-type E3s mediate functional changes in substrates via ubiquitin-like modification (e.g., TRIM38-induced SUMOylation). RING-type E3s stabilize substrates by changing the ubiquitylation types or removing ubiquitylation (e.g., TRIM44 and RNF26).

RING-type E3s-operated K63-linked polyubiquitylation is the most abundant linkage that activates signaling molecules. The proteins in the TRIM family, which comprises 82 members of the human genome, contain a conserved RING domain, one or two B-box domains, and a coiled-coiled domain (CCD). The C-terminal of TRIM proteins is a multifunctional domain and acts as a scaffold for protein-protein interactions, enzymatic activity, or nucleic acid binding [7]. In recent years, TRIMs have become the most detailed type of E3 that is capable of modulating host innate antiviral immune responses (Figure 2).

In the RLR pathway, RIG-I is autorepressed in physiological states and is activated after sensing virus infections. A model of the RIG-I activation process via E3-mediated sequential ubiquitylation has been proposed. The Riplet (also known as RNF135 or Reul) first ubiquitylates dsRNA-associated RIG-I at K788 to release the CARDs from autorepression [8,9]. The released CARDs are catalyzed into K63-linked ubiquitin chains at various sites. TRIM25 synthesizes K63-linked ubiquitin chains to decorate the RIG-I CARD on K172 [10,11]. TRIM4 is another E3 that modifies K63-linked polyubiquitin at the K164/172 of RIG-I to promote its activation and antiviral responses [12]. MEX3C (RNF194) also colocalizes with RIG-I and leads to its K63-linked ubiquitylation at the K48, K99, and K169 sites to enhance its antiviral responses, which is consistent with impairing IFN-β production in MEX3C KO mice [13]. However, in a HEK293T cell-free system knockout of the above E3s, the ubiquitylation of RIG-I does not require endogenous TRIM25, TRIM4, or MEX3C and is instead dependent on Riplet [14]. Unlike RIG-I, MDA5 is a protein with a linear structure, but it does have similar domains to RIG-I. TRIM65 has been confirmed as the major E3 that promotes the K63-linked polyubiquitylation of MDA5 at the K743 site to inhibit encephalomyocarditis virus (EMCV) infection [15]. MAVS is a major downstream signaling receptor for K63-ployubiquitylated RIG-I and MDA5 [16,17]. TRIM31 catalyzes the K63-linked polyubiquitylation of MAVS at the K10/311/461 sites, promotes its aggregation, and increases IFN-β production, thereby inhibiting virus infection [18]. As well as K63-linked polyubiquitylation, TRIM21-mediated K27-linked polyubiquitylation of MAVS also enhances innate immunity by increasing TBK1 binding to MAVS [19]. TRIM14 is an E3 localized to mitochondria and is a mitochondrial adaptor that recruits TRAF3/TBK1 and NEMO to facilitate RLR-mediated innate immune responses [20]. Under VSV infection, TRIM24 is transported to mitochondria and directly catalyzes the K63-linked polyubiquitylation of TRAF3 at the K429/436 sites while stabilizing the formation of the MAVS/TBK1 complex and the activation of the downstream antiviral signaling pathway [21]. TRIM44 contains an atypical Ub-binding zinc-finger UBP domain that enhances host antiviral responses by counteracting the proteasomal degradation from the K48-linked polyubiquitylation of MAVS [22]. The changes in these TRIMs due to highly pathogenic RNA viruses (such as SARS-CoV-2) require further study.

Laboratory of genetics and physiology 2 (LGP2) is another RLR proteins, which shares the conserved helicase and C-terminal domains with RIG-I and MDA5 but does not have a CARD domain. Although LGP2 is an RNA virus sensor, it cannot directly bind with and activate MAVS; however, it plays an important role in the positive and negative regulation of antiviral signaling and immune responses. New evidence has confirmed that LGP2 interacts with TRIM25 to prevent the TRIM25-mediated K63-linked polyubiquitylation of the RIG-I CARD domain [23]. Mechanically, LGP2 disrupts K63-linked ubiquitin chains from transforming into TRIM25 by interacting with Ubc13/UBE2N, which is a K63-Ub-conjugating enzyme from the E2 family. This interaction is also present in two other K63-linked E3s (TRAF6 and RNF125) to negatively regulate innate immune signaling [24,25]. However, accumulating evidence has shown that LGP2 can positively regulate MDA5-mediated antiviral signaling by synergistically sensing viral dsRNA [26,27]. However, it is unclear whether LGP2 affects the activation of MDA5 during the ubiquitylation process.

RNF128 binds to TBK1 and promotes TBK1 kinase activity by conjugating K63-linked polyubiquitin chains, thereby inducing IRF3 activation and IFN-β production [28]. Likewise, RNF41 also mediates the activation of TBK1 K63-linked polyubiquitylation to potentiate TRIF-dependent signaling [29]. However, RNF41 can also antagonize host antiviral responses in VSV-infected mice [30]. Therefore, RNF41 may be a multifunctional E3 in the RLR and TLR axes or it may affect species diversity. RING-UIM family member RNF166 interacts with TRAF3 and TRAF6 through its RING domain, enhances the ubiquitylation of TRAF3 and TRAF6 and also positively regulates RNA virus-triggered IFN-β production [31].

In the cGAS-STING-IRF3 axis, TRIM56 is first identified to interact with cGAS and promote its monoubiquitylation at the K335 site [32]. TRAF6 is a positive activator of cGAS via the promotion of its polyubiquitylation [33]. TRIM41, also known as the RING finger protein, interacts with C kinase (RINCK), binds to cGAS, and catalyzes its monoubiquitylation, thereby activating cGAS to synthesize cGAMP [34]. Compared to the direct activation of cGAS, TRIM14 indirectly increases cGAS stability by recruiting the deubiquitinase USP14 to cleave the K48-linked polyubiquitylation of cGAS [35]. Activated cGAS catalyzes the production of cGAMP from ATP and GTP, thereby forming homo- or heterodimers that bind and activate STING. TRIM56 and TRIM32 mediate the K63-linked polyubiquitylation of STING to form STING dimers, which are a prerequisite for TBK1 recruitment [36,37]. Furthermore, TRIM56 and TRIM32 are also essential for NF-κB activation and assure robust cytokine production in the cGAS-STING pathway. They directly catalyze the ubiquitylation activation of NEMO and subsequently activate IKKβ, which is required for TBK1 activation [38]. Activated TBK1 interacts with and then phosphorylates the 363LXIS366 motif in STING, which is a major IRF3-binding motif to promote the type I IFN production [39]. Hence, TRIM56 and TRIM32 positively regulated the cGAS-STING pathway via promoting the NEMO-IKKβ-TBK1 positive feedback loop.

In addition to the direct activation of the K63-linked polyubiquitylation of signaling molecules, several other mechanisms have also been confirmed. Firstly, RING-type E3s can catalyze other ubiquitin-type chains to activate or stabilize the protein. The TRIM23-catalyzed K27-linked polyubiquitylation of NEMO is also integrated to enhance innate RIG-I-, MDA5-, and TLR3-mediated signaling [40]. RNF185 specifically catalyzes the K27-linked polyubiquitylation of cGAS, thereby promoting its enzymatic activity [41]. RNF26 catalyzes the K11-linked polyubiquitylation of STING at the K150 site, which prevents the RNF5-mediated degradation of its K48-linked polyubiquitylation [42,43]. It also stabilizes STING and continuously activates antiviral immune responses. This also suggests that different E3s can catalyze different patterns of ubiquitylation by targeting the same sites in the same substrates, which leads to significant functional differences. Secondly, partial TRIMs stabilize and catalyze the activation of MAVS- or STING-recruited TBK1 to phosphorylate IRF3 for type I IFN production. A previous study found that mind bomb homolog 2 (MIB2), which is a RING-type E3, stabilized the MAVS-TBK1complex, depending on its interaction with the DLAIS motif in MAVS, which enhanced the K63-linked polyubiquitylation of TBK1 [44]. Thirdly, some RING-type E3s act as mediators to facilitate protein-protein interactions. TRIM9α, which is a short isoform of TRIM9, mediates GSK3β interactions and catalyzes TBK1 phosphorylation [45]. Auto-ubiquitylated TRIM26 also links TBK1 to NEMO, thereby promoting downstream signaling activation and limiting RNA virus infection [46].

### 3.2. Manipulation of RING-Type E3s That Suppresses the RLR and cGAS-STING Pathways

PRRs are key initiating molecules that recognize and bind PAMPs to initiate downstream signaling pathways and are tightly regulated to maintain moderate innate immune responses. In the RLR pathway, it is well known that the multiple mechanisms of RIGN-type E3s mediate RIG-I degradation but not MDA5 (Figure 2). After RNA virus infection, cytoplasmic c-Cbl (RNF55) is recruited by Siglec-G to form the SHP2/c-Cbl complex, which promotes the degradation of RIG-I through K48-linked polyubiquitylation at K813 [47]. Similar to the c-Cbl, RNF122 and RNF125 are also linked to the K48-linked polyubiquitylation to RIG-I and promote its degradation via proteasome [48,49]. Except for the classical K48-linked polyubiquitylation, TRIM40 binds directly to RIG-I and MDA5 and mediates their K27- and K48-linked polyubiquitin degradation via the UPP, thereby suppressing host antiviral signaling [50]. Furthermore, some RNA virus-encoded proteins also interfere with the stability of RIG-I via RING-type E3s, such as the porcine epidemic diarrhea virus (PEDV). Mechanistically, the stabilization mechanism of RIG-I is also mediated by FBXW7, which directly disrupts the SHP2/c-Cbl complex. FBXW7 promotes the K48-linked polyubiquitylation and degradation of SHP2 [51]. However, in PEDV-infected cells, PEDV nsp2 directly interacts with FBXW7 and promotes its degradation to disrupt the stabilization mechanism of RIG-I, thereby inhibiting the activation of host antiviral immune pathways [52]. Therefore, regulatory genes in E3s following virus infection should receive more attention in future studies.

Recently, TRIM7, also known as RNF90, has been shown to be an important negative regulator of the RLR and cGAS-STING pathways. Following RNA virus infection, TRIM7 specifically interacts with MAVS and promotes its K48-linked polyubiquitylation for proteasome-dependent degradation [53]. In DNA virus infection, TRIM7 tends to interact with STING, prompting its K48-linked ubiquitylation to initiate proteasome-dependent degradation [54]. Likewise, the alveolar macrophage-specific TRIM29 and mouse-specific TRIM30α promote the K48-linked polyubiquitylation of STING, thereby promoting its proteasome-dependent degradation [55,56,57,58]. Except for directly degradated STING, STING also recruits TRIM21 to degrade DNA sensors, such as DDX41 and IFI16. Mechanistically, TRIM21 can promote the K48-linked polyubiquitylation of DDX41 at the K9 and K115 sites, leading to its degradation and reducing the production of type I IFN [59]. STING also recruits TRIM21 to promote IFI16 degradation via the UPP [60]. The abovementioned studies have also explored several negative loop mechanisms of host-sensing pathogens.

Several TRIMs were also recruited to negatively regulate the activation or stability of TBK1 and IRF3/7 during virus infection (Figure 2). TRIM11 also interacts with TBK1 and is thought to block IRF3-mediated IFN-β production [61]. In cells infected by SeV and VSV, TRIM26 can be introduced into the nuclei. In those nuclei, TRIM26 interacts with phosphorylated IRF3 and promotes its K48-linked polyubiquitination and proteasomal degradation [62]. RNA (SeV and VSV) or DNA (HSV) virus infections upregulate Siglec1 expression in macrophages and indirectly recruit TRIM27 to mediate the Ub-dependent degradation of TBK1 [63]. In addition, the deubiquitinase USP7 can maintain stability by cleaving the K48-linked polyubiquitylation chains in TRIM27, thereby potentiating TRIM27-mediated TBK1 degradation [64]. The TRIM27-TBK1-USP7 constructs a type I IFN signaling feedback loop that adapts to different cellular states after viral infection.

IRF3 acts as a transcription factor that generates type I IFN production in the early phases of virus infection. FBXO6 and FBXO17 have been demonstrated to have novel atypical mechanisms by which F-box proteins regulate IRF3 degradation [65,66]. They interact with the IAD domain of IRF3 independently of the F-box domain. FBXO17 sequesters PP2A and reduces IRF3 dimerization and nuclear translocation [67]. A recent study on zebrafish suggested that both IRF3 and IRF7 are decorated with K27-linked polyubiquitylation chains to facilitate their degradation by FBXO3 [68]. However, whether this degradation process is dependent on the SKP1-Culs complex and whether it exists in mammals is unclear. Furthermore, canonical SCF E3s specifically recognize and bind to phosphorylated proteins. The relationship between phosphorylated IRF3/7 and FBXO3 in degradation mechanisms requires future study. Recently, MEKK2 from tumor-derived exosomes has been shown to phosphorylate IRF3, which then promotes IRF3 polyubiquitylation and blocks its dimerization, nuclear translocation, and transcriptional activity following virus infection [69]. Therefore, whether MEKK2 promotes IRF3 degradation through SCFFBXO3 also requires further investigation.

## 4. Manipulation of HECT-Type E3s in the RLR and cGAS-STING Pathways

**Figure 3:**
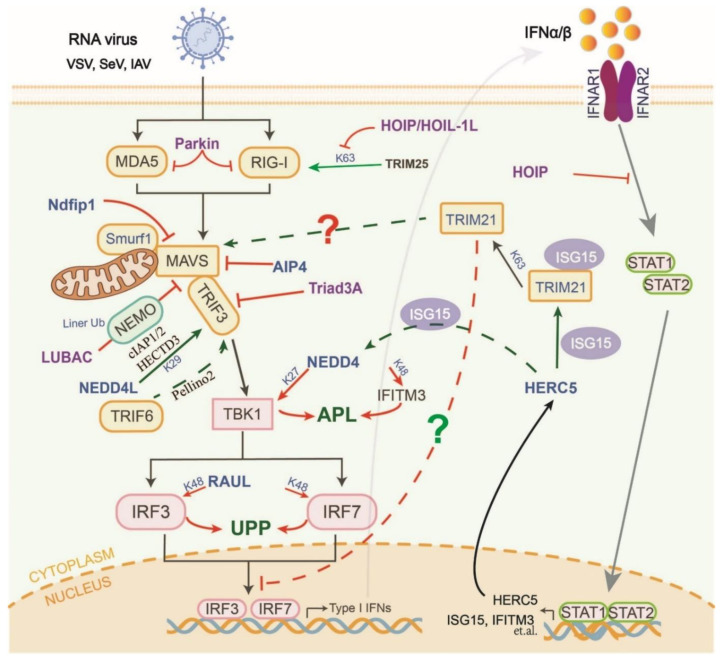
The members and mechanisms of HECT- and RBR-type E3s that regulate RLR and cGAS-STING signaling pathways. HECT-type E3s mainly mediate protein degradation via the UPP or ALP to inhibit the activation of RLR (e.g., RAUL, NEDD4, Parkin, etc.). On the other hand, HECT- and RBR-type E3s indirectly regulate the activation of the pathways by targeting other E3 enzymes (e.g., HOIP/HOIL-1L and HERC5). In particular, the relationship between HERC5-mediated ISGylation and RLR signaling axis needs further study. RBR-type E3s are also involved in regulating downstream IFN signaling pathways (e.g., HOIP). The solid line represents the direct regulation effect (arrow: positive regulation; short line: negative regulation), the dotted line represents the indirect regulation effect; the question mark represents no experimental verification.

The Nedd4-like family has nine members in humans. NEDD4 is a multifunctional regulator of host antiviral innate immune responses (Figure 3). NEDD4 contains a conserved C2 domain, four tryptophan–tryptophan (WW) domains, and a C-terminal HECT domain [70]. It was first identified that NEDD4 directly targets TBK1 to induce K27-linked polyubiquitylation at K344 for degradation via the ALP, leading to the downregulation of type I IFN signaling in the late stages of virus infection [71]. Moreover, NEDD4 can also interact with the PPxF motif in IFITM3 to promote its K48-linked polyubiquitylation and subsequent lysosomal degradation, thereby inhibiting host antiviral responses [72]. In addition to suppressing host innate antiviral immune responses, NEDD4 also directly recognizes the PPxY domain of multi-enveloped viruses via its third WW domain (WW3), which completes the germination process of progeny virions [73]. Recently, NEDD4 has been shown to have positive effects on several viral infections, including SARS-CoV-2 and the Ebola virus [74]. Therefore, highly potent inhibitors that target NEDD4 may be used to develop novel host-oriented broad-spectrum antiviral drugs.

Unlike NEDD4, Ndfip1 is a recruiter and activator of multiple HECT-type E3s from the Nedd4-like family. Ndfip1 enhances the auto-ubiquitylation of Smurf1 and its interaction with MAVS, which ultimately promotes the degradation of MAVS [75]. Furthermore, another HECT-type E3 (AIP4) is recruited by PCBP2 to promote the degradation of MAVS following virus infection [76]. The PCBP2-AIP4 axis can be hijacked by SARS-CoV ORF-9b to trigger the degradation of MAVS, TRAF3, and TRAF6 [77]. HECT-type E3 RAUL reduces the production of type I IFNs through direct interactions with IRF3 and IRF7 and catalyzes their K48-linked polyubiquitylation, which is then degraded via the UPP [78].

Unlike NEDD4, NEDD4L is an important E3 switch that actively induces the production of type I IFN and proinflammatory cytokines following virus infection (Figure 3). Mechanistically, NEDD4L directly interacts with TRAF3 and catalyzes its K29-linked ubiquitylation at the Cys56 and Cys124 sites. Ubiquitylated TRAF3 further interacts with other E3 ligases (e.g., cIAP1/2 and HECTD3), resulting in enhancing TRAF3 activation and type I IFN production [79]. This process may depend on the activation of NEDD4L by Pellino2 and TRAF6 [80]. The details of their regulation of virus infection warrant deeper research.

## 5. Manipulation of RBR-Type E3s in the RLR and cGAS-STING Pathways

The linear Ub assembly complexes (LUBACs), consisting of three E3 ligases (HOIL-1L, HOIP, and the accessory protein SHARPIN), are multifunctional in regulating host antiviral responses (Figure 3). HOIP and HOIL-1L independently interact with TRIM25 and RIG-I to negatively regulate virus-induced type I IFN production in a mechanistically dissociated manner. On the one hand, the HOIP/HOIL-1L LUBAC promotes the ubiquitylation and degradation of TRIM25 [81]. On the other hand, the NZF domain of HOIL-1L competes with TRIM25 to interact with RIG-I. The mechanism described above specifically initiates the K63-linked polyubiquitylation and activation of RIG-I [81]. Furthermore, LUBACs can also bind to NEMO and catalyze its linear ubiquitylation in the late stages of viral infection. Linear ubiquitylated NEMO competes with MAVS to bind to TRAF3, thereby disrupting the MAVS-TRAF3 complex and type I IFN production. Simultaneously, it stimulates the action of NF-κB-dependent signaling [82]. Triad3A, which is a member of the RBR family, targets TRAF3 for K48-linked polyubiquitylation and degradation following RNA virus infection and negatively regulates RLR signaling [83].

Recent studies have shown that virus infection can induce HOIP expression via the NF-kB pathway. HIOP then interacts with STAT1 and catalyzes its linear ubiquitylation at the K511 and K652 sites, which in turn disrupts the binding of STAT1 to the interferon receptor IFNAR2 [84]. This suggests a transition mechanism between the homeostasis and activation of type I IFN signaling.

## 6. Manipulation of Atypical E3s in the RLR and cGAS-STING Pathways

In addition to typical E3 activity, some E3s exhibit SUMO ligase activation and the regulation of the RLR and cGAS-STING axis (Figure 2). TRIM28 mediates the SUMOylation of IRF7 to induce transcriptional repression [85]. TRIM38 has recently been proven to stabilize RIG-I and MDA5 against their K48-linked polyubiquitylation-dependent degradation in uninfected cells or cells in the early stages of infection [86]. Moreover, during the early phases of DNA virus infection, TRIM38 catalyzes the SUMOylation of cGAS and STING to prevent their K48-linked polyubiquitylation and subsequent degradation via the UPP and chaperone-mediated autophagy pathways, respectively [87]. Notably, mTRIM38 has been shown to first SUMOylate cGAS (K217 and K464) and then regulate its subsequent K48-linked polyubiquitylation and degradation [87]. The abovementioned studies have indicated that TRIM38 has obvious differences in function between species.

ISGylation is a multistep process involving IFN-inducible Ube1L, UbcH8, and E3s that bind interferon-stimulated gene 15 (ISG15) to substrate proteins. Recently, HERC5/Cebl, an IFN-induced HECT-type E3, has been identified as being involved in E3-mediated ISGylation (Figure 3). HERC5 enhances the activation of TRIM21 by catalyzing the ISGylation at its Lys260 and Lys279 residues. ISGylated TRIM21 then induces K63-linked polyubiquitylation of p62, which prevents its tendency to auto-oligomerize and target autophagosomes [88]. However, the relationship between the HERC5-TRIM21-p62 axis and innate antiviral immune responses remains unclear. TRIM21 can activate MAVS to enhance its antiviral activity [19]. Additionally, p62 is a major receptor for ubiquitylated proteins and is involved in the negative regulation of RLRs via the autophagy pathway [89,90]. Therefore, the HERC5-TRIM21-p62 axis may be involved in self-positive regulatory mechanisms in innate immune systems by maintaining the stability and antiviral activity of important proteins (Figure 3). Additionally, NEDD4 is negatively regulated by ISG15. Free ISG15 specifically binds to NEDD4 and blocks its interaction with the Ub-E2 molecule, thus preventing the transfer of Ub from E2 to E3s [91]. The ISGylation mechanism of NEDD4 is currently unknown. As it is the only identified ISGlyation-related E3s, whether HERC5 uses NEDD4 as a substrate may require further studies to confirm.

## 7. Manipulation of E3s-Mediated Autophagy in the RLR and cGAS-STING Pathways

**Figure 4:**
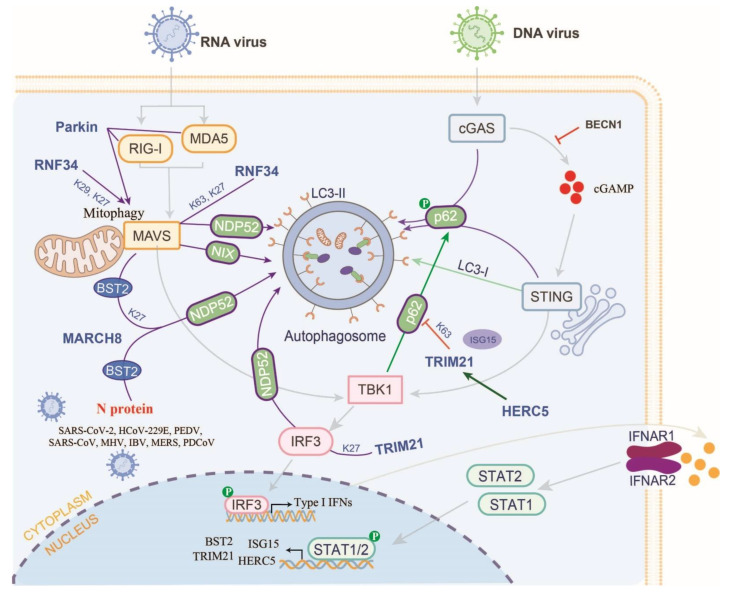
E3s are hijacked to regulate host antiviral responses via selective autophagy. E3s catalyze the ubiquitylation of substrates that are to be bound by autophagy receptors (e.g., NDP52 and p62) and then degrade the substrates via the ALP (e.g., RNF34 and TRM21). E3s also promote the occurrence of mitochondrial autophagy to degrade the mitochondrial protein (e.g., RNF34 and Parkin). except for host protein, E3s also directly or indirectly target ubiquitin virus proteins that interact with autophagy receptors and then degrade via the ALP (e.g., RNF34). HERC5 can promote the ISGylation of TRIM21 to inhibit the K63-linked polyubiquitylation of p62, thereby suppressing the ALP.

Ub-induced selective autophagy is another pathway for protein degradation besides the E3s-induced protein degradation via UPP. To date, a total of seven cargo receptors that bind to ubiquitylated proteins have been identified, including SQSTM1/p62, CALCOCO2/NDP52, optineurin (OPTN), neighbor of BRCA1 (NBR1), BNIP3L, TOLLIP, and TAX1BP1 [92]. However, only the mechanisms by which only p62 and NDP52 bind to different proteins and mediate RLR and cGAS-STING axis activation have been well defined (Figure 4). The functions of other autophagy receptors may require more attention in terms of understanding how they regulate the host innate antiviral immune response.

In the RLR, RIG-I activation has been shown to invoke autophagy and be dependent on the MAVS-TRAF6-Beclin-1 axis [93]. Furthermore, the RBR-type E3 Parkin directly interacts with RIG-I and MDA5 and subsequently catalyzes their K48-linked polyubiquitylation and degradation by mediating mitophagy [94]. Recently, a truncated form of MAVS has been developed that allows full-length MAVS but avoids aberrant aggregation under normal physiological conditions. Abnormal MAVS aggregates are tagged and bind to the autophagy receptor NIX, thereby promoting mitophagy and degradation [95]. RNF34 catalyzes the K27- and K63-linked polyubiquitylation of MAVS at the K311 site, thereby facilitating the NDP52-mediated autophagic degradation of MAVS. Furthermore, RNF34 also catalyzes the K27- and K29-linked polyubiquitylation of MAVS at the K297/311/348/362 sites and clears mitochondria damaged by virus infection [96]. Therefore, it is difficult to determine whether the degradation of RIG-I/MAD5 and MAVS is dependent on mitochondrial fragmentation or mitophagy.

MARCH8, also known as RNF178, has turned out to be a multifunctional E3 that mediates the RLR axis and controls coronavirus infection. Firstly, MARCH8 is recruited by Tetherin (BST2/CD317) and interacts with MAVS following SeV infection, thereby promoting the K27-linked polyubiquitylation of MAVS. Ubiquitylated MAVS is then targeted by the cargo receptor NDP52 for degradation via ALP [97]. As well as promoting the degradation of MAVS, MARCH8 induces the degradation of various coronavirus N proteins, thereby inhibiting viral replication. Mechanistically, MARCH8 is recruited by PABPC4 to catalyze the N protein ubiquitylation of eight CoVs, including SARS-CoV-2, HCoV-229E, PEDV, SARS-CoV, MHV, IBV, MERS, and PDCoV. Ubiquitylated CoV N proteins are targeted by the cargo receptor NDP52/CALCOCO2 and degraded by the ALP to inhibit COV replication [98]. Moreover, PEDV infection upregulates the expression of early growth response gene 1 (EGR1). EGR1 facilitates the expression of IFN-regulated antiviral (IRAV), which directly interacts with the PEDV N protein and catalyzes its ubiquitylation by MARCH8 [99]. Hence, following CoV infection, MARCH8 can inhibit the intracellular proliferation of coronavirus by preferentially degrading virus proteins and maintaining the stability of MAVS and its activated antiviral IFN production. Since it is unclear whether MARCH8 degrades MAVS under different coronavirus infection conditions, more experimental data are needed to confirm this conclusion.

The cGAS-STING signaling pathway is also regulated by autophagy (Figure 4). In DNA virus-infected cells, the activated cGAS-STING pathway can recruit TBK1 to phosphorylate IRF3, thereby activating type I IFN production. On the other hand, TBK1 also phosphorylates p62/SQSTM1, which targets cGAS and STING and promotes autophagic degradation [100]. The STING targeting of cGAMP is transferred from the ER to the ERGIC and Golgi apparatus. This promotes the process of autophagy by constructing membranes for LC3 lipidation [101]. Another study also showed that cGAS interacted with BECN1 to suppress the synthesis of cGAMP and avoid the continuous progress of immune stimulation [102]. Additional regulatory pathways may exist to promote the autophagic degradation of cytoplasmic DNA or alter signaling following DNA virus infection.

TBK1 and IRF3 are common downstream molecules in the RLR and cGAS-STING pathways, and their activation is also regulated by E3s-mediated autophagic degradation. TRIM23 is essential for virus-induced autophagy to modulate host innate immunity. Mechanistically, TRIM23 auto-ubiquitylates itself with K27-linked polyubiquitylation chains to interact with and activate TBK1, thereby facilitating the phosphorylation of the cargo receptor p62 [103]. A recent study reported that TRIM21 could promote the K27-linked polyubiquitylation of IRF3 in a CALCOCO2/NDP52-dependent manner, thereby inducing its autophagic degradation [104]. The deubiquitinase PSMD14 stabilizes IRF3 by removing its K27-linked polyubiquitylation chains [104]. In this case, the CALCOCO2/NDP52-dependent autophagic degradation of IRF3 can be counteracted by PSMD14, which positively regulates the host antiviral responses.

## 8. Concluding Remarks and Future Directions

E3s play a key role in Ub-mediated host antiviral innate immune responses. Some E3 members catalyze the host’s natural immune response and improve the host’s antiviral capabilities. Some E3s use negative regulatory mechanisms to avoid excessive immune responses, such as inflammation. The currently known mechanisms of E3s-mediated host virus responses include ubiquitylation and SUMOylation, catalyzed protein activation, and the stabilization of the UPP or ALP. Recently, Neddylation and UFMylation, depending on RNF111 and a novel type-E3 ligase (UFL1), have been identified as mediating cGAS and RIG-I activation [105,106]. Furthermore, myeloid neddylation targets and active IRF7 are also necessary for host innate immunity against RNA viruses [107]. However, it is unclear whether and which E3s are involved in this process. On the other hand, PTMs from E3-mediated substrates also communicate with other types of PTMs, such as phosphorylation, acetylation, etc. Therefore, more research should be carried out in the future to reveal the crosstalk and mechanisms between different PTMs.

E3s have been considered as potential drug targets that directly enhance host innate antiviral immune responses. Moreover, there are several E3s that specifically function to degrade virus proteins. Proteolysis-targeting chimeric (PROTAC) technology offers a technique for rapidly and reversibly inducing protein degradation in vivo, such as the auxin-inducible degron (AID) [108]. PROTAC has been successfully applied to degrade virus proteins from IAV via the endogenous UPP of host cells [109]. The mechanisms of innate immune responses are tightly regulated by the selective expression of innate immune factors and the PTMs of key enzymes in host tissues. With the development of this technology, the application prospects of E3s in the direct targeted therapy of viruses could become even broader. Nevertheless, several key issues still need to be addressed before they can be used in clinical treatments. For example, the correspondence between E3s and specific viral infections is unclear, whether E3s have cell-specific or tissue-specific immunomodulatory effects remains unclear, and the system that efficiently and stably modulates the angle switch of degron also needs more study. In addition, homologous or heterologous E3 can act as substrates for each other, thereby directly or indirectly modulating the state of host innate antiviral immunity [81,88,110]. A more in-depth study on the interactive regulatory networks among E3s is required.

In conclusion, this review demonstrates that E3s, as a ubiquitylated operon, play key roles in regulating host innate antiviral immune response, especially in the balance of innate immune responses via viral sequestration. It is also worth noting that E3s upregulate or downregulate the activity of specific members in the interferon signaling pathway through ubiquitylation modifications. In turn, various interferon-inducible genes could affect E3s, thereby forming a feedback regulatory loop mechanism. Therefore, elucidating the mechanisms of E3s in host innate immune responses and viral infections seems essential for providing a comprehensive rationale for the development of new therapeutic strategies.
